# Polysomnographic Sleep Is Associated With Time to Develop Dementia: A Study Using 19-Year VA National EHR Data

**DOI:** 10.1093/geroni/igaa057.1520

**Published:** 2020-12-16

**Authors:** Sara Nowakowski, Javad Razjouyan, Amir Sharafkhaneh, Mark Kunik, Aanand Naik

**Affiliations:** 1 Baylor College of Medicine, Houston, Texas, United States; 2 Center for Innovations in Quality, Effectiveness, and Safety (iQuest), Houston, Texas, United States; 3 Michael E. DeBakey VA Medical Center, houston, Texas, United States

## Abstract

Few studies have longitudinally investigated the association between objectively measured sleep and time to develop dementia. This study leverages polysomnography (PSG) sleep data extracted from the VA national electronic health records (VA-EHR) to assess the association between sleep and time to develop dementia. We identified 61,165 PSG reports from the VA-EHR from 2000 to 2019 using CPT codes. Patients who developed dementia were identified using all-cause dementia ICD-9/10 codes documented on two separate visits starting one year after the PSG study until the end of 2019 in a 1-year sliding period (n=1,534). Using the first appearance of ICD-9/10 code as dementia onset time, patients were clustered into 3 groups of early-, mid-, and late time to develop dementia (mean = 2.7, 7.5, 12.8 years, respectively). Natural language processing was used to extract sleep efficiency (SE) and sleep onset latency (SOL). Univariate analysis was used to compare the groups. After adjusting for age, SE was significantly higher in the late (76%) vs early (69%) group and SOL was significantly shorter in late (21m) versus early (33m) group. SE was higher and SOL was shorter in patients who developed dementia later compared to those who developed dementia earlier. Greater sleep continuity in late dementia onset group suggests that sleep may be a modifiable risk factor that could potentially delay the onset of dementia.

